# Prognostic significance of *PIK3CA* mutation in stage IIB to IVA cervical cancers treated by concurrent chemoradiotherapy with weekly cisplatin

**DOI:** 10.1097/MD.0000000000011392

**Published:** 2018-08-03

**Authors:** Bouchra Lachkar, Takeo Minaguchi, Azusa Akiyama, Shuling Liu, Shuang Zhang, Chenyang Xu, Ayumi Shikama, Nobutaka Tasaka, Manabu Sakurai, Sari Nakao, Hiroyuki Ochi, Hiroyuki Yoshikawa, Toyomi Satoh

**Affiliations:** a Department of Obstetrics and Gynecology, Faculty of Medicine; b Doctoral Program in Obstetrics and Gynecology, Graduate School of Comprehensive Human Sciences, University of Tsukuba, Tsukuba; c Ibaraki Prefectural Central Hospital, Ibaraki, Japan.

**Keywords:** cervical cancer, chemoradiotherapy, cisplatin, mutation, PIK3CA

## Abstract

The standard treatment for locally advanced cervical cancer is cisplatin-based concurrent chemoradiotherapy (CCRT). Although the activated PI3-kinase/Akt pathway is known to be involved in both cisplatin-resistance and radioresistance, to date, only a few studies have reported significant associations between *PIK3CA* gene mutational status and outcome by CCRT in the disease. The aim of this study was to clarify the prognostic significance of *PIK3CA* mutational status in cervical cancers treated by CCRT.

We analyzed *PIK3CA* mutation in 59 patients with stage IIB to IVA cervical carcinomas primarily treated by CCRT with weekly cisplatin using formalin-fixed paraffin-embedded biopsy specimens before treatment. Fifty-seven of 59 patients (97%) had locally advanced cancers with stage IIIA to IVA. Clinicopathologic data and patient survival were retrospectively compared according to *PIK3CA* mutational status.

*PIK3CA* mutation was found in 7 of 59 patients (12%). No significant differences in clinicopathologic characteristics were observed according to *PIK3CA* mutational status. Patients with wild-type *PIK3CA* showed significantly improved cancer-specific survival as compared with mutated patients (*P* = .044). Subsequent survival analyses revealed that *PIK3CA* mutation was a significant prognostic factor for poor overall survival [multivariate adjusted hazard ratio (HR), 3.9; 95% confidence interval (95% CI), 1.3–11.8; *P* = .017] and cancer-specific survival (multivariate adjusted HR, 3.6; 95% CI, 1.2–11.0; *P* = .024).

Together with previous published findings, the current study further supports the clinical significance of *PIK3CA* mutation in cervical cancer. Our observations suggest that molecular inhibitors targeting the PI3-kinase/Akt pathway may improve the outcome by CCRT in cervical cancers harboring *PIK3CA* mutation, providing significant implications for novel treatment strategy based on precision medicine in the disease.

## 1 Introduction

Incidence and mortality of cervical cancer are both the 4th in women worldwide, and cervical cancer causes 7.5% of all female cancer deaths (CANCER TODAY; http://gco.iarc.fr/today/home). Although current standard treatment for locally advanced cervical cancer is cisplatin-based concurrent chemoradiotherapy (CCRT), the 5-year survival rate in advanced cervical cancer patients is still low (30–50%). Hence, the development of novel treatment strategy to improve outcome by CCRT is urgently required. *PIK3CA* mutation is reportedly the most common genetic alteration in cervical cancers, followed by *KRAS*, *EGFR*, and then PTEN loss.^[1]^ Thirteen to 36% of cervical cancers are reported to harbor *PIK3CA* mutations.^[2]^*PIK3CA* gene encodes p110α protein, the catalytic subunit of phosphoinositide 3-kinase (PI3K), and is known to be mutated or amplified in many kinds of human cancers. The PI3K/Akt signaling is one of the pivotal pathways for human carcinogenesis. Binding of growth factors phosphorylates and activates the tyrosine kinase receptor on cell membrane. The active receptor then turns on the PI3K enzyme attached at its bottom, and PI3K phosphorylates phosphatidylinositol-4,5-bisphosphate (PIP2) to phosphatidylinositol-3,4,5-triphosphate (PIP3). PIP3 activates phosphoinositide-dependent kinase 1 (PDK1) enzyme that will phosphorylate and activate Akt. The activated Akt will then regulate downstream components necessary for a variety of cellular functions, including cell cycle, apoptosis, protein synthesis, DNA damage repair, and angiogenesis. Besides, the PI3K/Akt pathway is known to be involved in resistance to cisplatin. Akt is reported to induce cisplatin resistance through inhibiting the downstream proteins such as p21, Mdm2, Bad, Bax, and Caspases.^[3]^ The PI3K/Akt pathway is also known to be involved in radioresistance through the following mechanisms.^[4]^ The PI3K/Akt pathway regulates DNA-dependent protein kinase (DNA-PK), which is responsible for the repair of DNA double-strand breaks caused by irradiation. In addition, inhibiting the PI3K/Akt pathway downregulates hypoxia-inducible factor 1-α and vascular endothelial growth factor, resulting in normalized vasculature and decreased hypoxia. However, to date, only a few studies have reported the prognostic significance of *PIK3CA* mutation in cervical cancers treated by CCRT. McIntyre et al^[5]^ reported that overall survival (OS) at 5 years after CCRT was significantly worse for *PIK3CA* mutant patients compared with *PIK3CA* wild-type patients (40% vs 70%). In another study conducted by Wang et al,^[2]^ patients with *PIK3CA* mutations had a significantly lower complete response rate to CCRT, 52% against 86% in *PIK3CA* wild type. Further accumulation of evidence is warranted to clarify the prognostic significance of *PIK3CA* mutation for the outcome after CCRT. Hence, the aim of the current study was to clarify the prognostic significance of *PIK3CA* mutational status in cervical cancers treated by CCRT with weekly cisplatin. We demonstrate here that *PIK3CA* mutation was a significant prognostic factor for poor OS and cancer-specific survival (CSS) after CCRT. Our findings provide significant implications for a novel treatment strategy for cervical cancer.

## 2 Materials and methods

### 2.1 Patients and treatment

Patients with cervical carcinomas who were primarily treated by CCRT with weekly cisplatin at the University of Tsukuba Hospital between 2001 and 2015 were identified through our database, and their medical records were retrospectively reviewed. Staging at diagnosis was performed on the basis of the International Federation of Gynecology and Obstetrics (FIGO) system. Patients mostly received whole pelvic irradiation of 50 Gy and brachytherapy of 24 Gy concomitant with 5 to 6 cycles of weekly administration of 40 mg/m^2^ cisplatin. All samples were obtained with informed consent or opt-out procedure in accordance with protocols approved by the Ethics Committee University of Tsukuba Hospital. Median follow-up duration was 77 months. Follow-up data were retrieved until June 30, 2017. Table 1 summarizes patient characteristics.

**Table 1:**
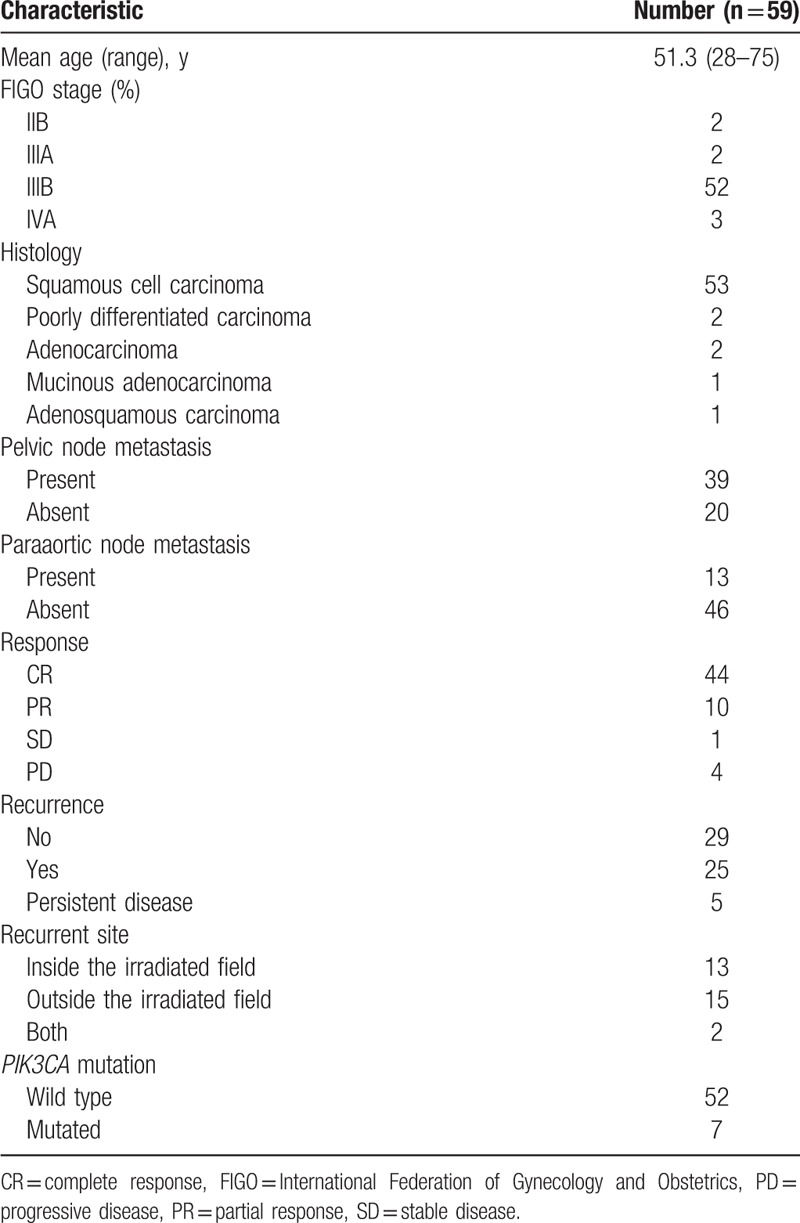
Patient characteristics.

### 2.2 DNA extraction and *PIK3CA* mutation analysis

Genomic DNAs were extracted from formalin-fixed, paraffin-embedded (FFPE) biopsy specimens before treatment using blackPREP DNA Kit (GenoStaff, Tokyo, Japan) according to the manufacturer's instruction. Mutation analysis of *PIK3CA* gene was performed as described previously.^[6]^

### 2.3 Statistical analyses

Differences in proportions were evaluated by the Fisher exact test. Differences in continuous variables were evaluated by the *t* test. Kaplan–Meier survival curves were calculated and compared statistically using the log-rank test. The Cox proportional hazard model was used for univariate analysis and, after adjustment for baseline characteristics and prognostic factors (age, FIGO stage, histology, and pelvic node metastasis), multivariate analysis.

## 3 Results

**Figure 1:**
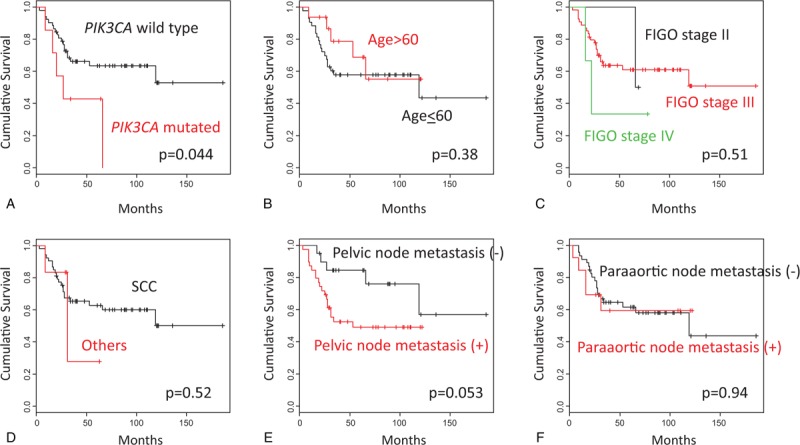
Kaplan–Meier curves for cancer-specific survival in cervical cancers treated by CCRT. (A) Cases with wild-type *PIK3CA* (n = 52) versus mutant *PIK3CA* (n = 7); (B) Cases with age ≤ 60 years (n = 43) versus age > 60 years (n = 16); (C) Cases with FIGO stage II (n = 2) versus III (n = 54) versus IV (n = 3); (D) Cases with squamous cell carcinomas (n = 53) versus other histologic types (n = 6); (E) Cases with negative pelvic node metastasis (n = 20) versus positive pelvic node metastasis (n = 39); (F) Cases with negative paraaortic node metastasis (n = 46) versus positive paraaortic node metastasis (n = 13).

**Figure 2:**
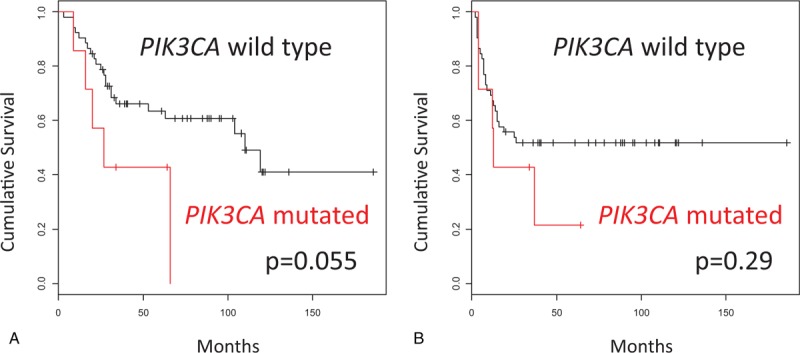
Kaplan–Meier curves in cervical cancers treated by CCRT. (A) Overall survival in cases with wild-type *PIK3CA* (n = 52) versus mutant *PIK3CA* (n = 7); (B) Progression-free survival in cases with wild-type *PIK3CA* (n = 52) versus mutant *PIK3CA* (n = 7).

We analyzed DNA sequences on exons 9 and 20 of the *PIK3CA* gene in archival FFPE biopsy specimens before treatment by direct sequencing in 59 patients with stage IIB to IVA cervical carcinomas treated by CCRT with weekly cisplatin. Among the patients, 57 (97%) were locally advanced cancers (Table 1). We found *PIK3CA* mutation in 7 of 59 patients (12%). Five (71%) of the mutations were mapped on the helical domain, and 1 (14%) on the kinase domain of the p110α protein (Table 2). We next compared various clinicopathologic features according to *PIK3CA* mutational status, finding no significant differences in any of the variables, including age, FIGO stage, histologic subtype, and response rates after CCRT (Table 3). Subsequently, we compared CSS according to various prognostic factors including *PIK3CA* mutational status (Fig. 1). Pelvic node metastasis showed a difference without statistical significance (*P* = .053; Fig. 1E). Interestingly, *PIK3CA* mutation was the only prognostic factor showing a significant association with poor CSS (5-year survival rate: 64% vs 43%; *P* = .044; Fig. 1A). We also compared OS and progression-free survival (PFS) according to *PIK3CA* mutational status, but both differences were not significant (5-year survival rate: 64% vs 43% and 52% vs 21%; *P* = .055 and .29, respectively; Fig. 2A, B). Finally, we conducted univariate and multivariate analyses of *PIK3CA* mutation for survival (Table 4). Notably, adjusted multivariate analysis demonstrated that *PIK3CA* mutation was significant for poor OS [hazard ratio (HR), 3.9; 95% confidence interval (95% CI), 1.3–11.8; *P* = .017; Table 4] and CSS (HR, 3.6; 95% CI, 1.2–11.0; *P* = .024; Table 4), but not for poor PFS (HR, 2.3; 95% CI, 0.81–6.5; *P* = .12; Table 4).

**Table 2:**
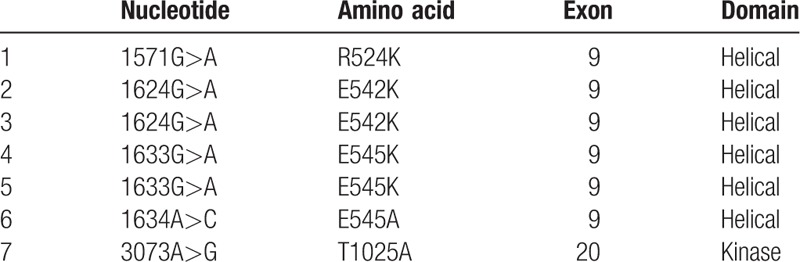
Results of *PIK3CA* mutation analysis.

**Table 3:**
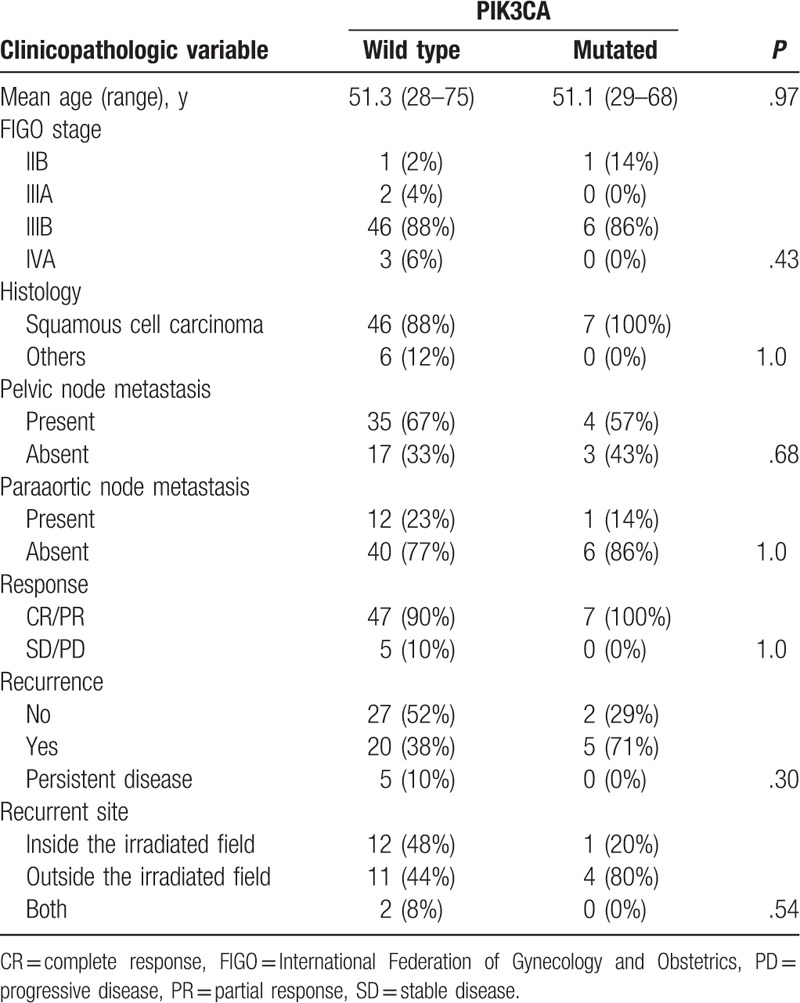
Relationship between *PIK3CA* mutational status and clinicopathologic variables.

**Table 4:**
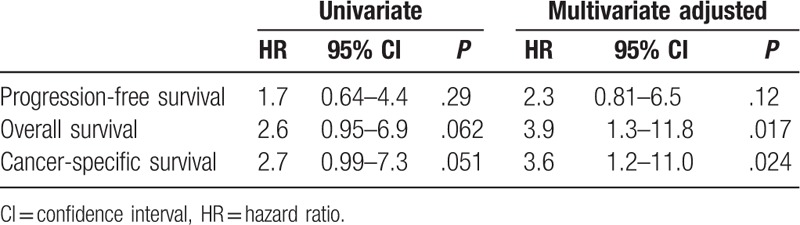
Univariate and adjusted multivariate analyses of *PIK3CA* mutational status for survival.

## 4 Discussion

Our mutational analysis found *PIK3CA* mutations in 12% of the patients, which is relatively lower than the results previous reported (13–36%;^[2]^), possibly due to difference in quality of samples for DNA extraction, as we used archival FFPE biopsy specimens from patients with mostly locally advanced cancers before treatment. PI3K is a heterodimer of the catalytic subunit p110α and the regulatory subunit p85α. P110α is composed of 5 domains: an adaptor-binding domain (ABD; residues 16–105), a Ras-binding domain (RBD; residues 187–289), a C2 domain (residues 330–487), a helical domain (residues 517–694), and a kinase domain (residues 797–1068). P85α also comprises 5 domains: an SH3 domain, a GAP domain, an N-terminal SH2 (nSH2) domain, an inter-SH2 domain (iSH2), and a C-terminal SH2 domain (cSH2). All of the mutations found in our study were mapped inside the helical or kinase domains of p110α (Table 2). E542 and E545 on the helical domain are suggested to biochemically interact with K379 and R340 of the nSH2 of p85.^[7,8]^ Moreover, the crystal structure of p110α/p85α complex reportedly showed that E542 and E545 are located at the interface with nSH2 in close proximity to the nSH2-kinase domain interface, suggesting a mechanism whereby E542K and E545K mutations can affect the enzyme activity of p110α.^[9]^ T1025 was shown to be located close to the N-terminus of the catalytic loop, and may therefore alter the enzyme activity through changing the conformation of the catalytic loop.^[9]^ On the basis of these structural information, we regard that considerable translational significance lies in our subsequent analyses on the associations between *PIK3CA* mutational status and clinicopathologic data.

Our subsequent survival analyses revealed that patients with mutant *PIK3CA* had significantly worse CSS than those with wild type, and that *PIK3CA* mutation was a significant prognostic factor for poor OS and CSS. As regards the prognostic impact of *PIK3CA* mutation, McIntyre et al ^[5]^ previously reported that *PIK3CA* mutational status was strongly associated with OS in FIGO stage IB/II patients, but not in stage III/IVA patients (*P* = .0002 vs *P* = .98). However, most of the patients in our study were stage III/IVA (97%; Table 1). Likewise, Wang et al^[2]^ reported that patients without genetic alterations (mutations or amplification) of *PIK3CA* had a significantly higher response rate than those with the alterations (*P* = .006); however, our study did not find any difference in the response rate (Table 3). These discrepancies among studies may be attributed to differences in analyzed genetic alterations (including or not amplification), constitution of histological subtypes (including or not other than SCC) and FIGO stages, patient follow-up durations, and/or recurrence treatment strategies. In any case, the current findings further support the significance of *PIK3CA* genetic aberrations on outcome of cervical cancers treated by CCRT.

The results of our survival analyses are suggestive of a possibility that inhibiting PI3K by molecular targeting agents may improve outcome by CCRT with cisplatin. Regarding the effect of PI3K inhibitor combined in the treatment of cervical cancer, there have been some preclinical studies reported. Xie et al ^[10]^ have recently reported that a dual PI3K/mTOR inhibitor NVP-BEZ235 treatment in combination with cisplatin or carboplatin induced a synergistic antitumor response in cervical carcinoma cells in vitro. Likewise, PI3-kinase inhibitor LY294002 reportedly radiosensitized cervical cancer cell lines in vitro^[11]^ and in vivo.^[12]^ Moreover, Arjumand et al^[13]^ recently examined in vitro whether mutated *PIK3CA* confers cervical cancer cells higher resistance to cisplatin and/or radiation, and whether this phenotype is reversed by inhibiting PI3K. They reported that CaSki cells harboring heterozygous E545K were more resistant to cisplatin/cisplatin + radiation than HeLa or SiHa cells with wild-type *PIK3CA*, and that HeLa cells stably expressing E545K were more resistant to cisplatin/cisplatin + radiation than cells with wild-type/depleted *PIK3CA*. Cells expressing E545K showed constitutively activated PI3K pathway and augmented cell migration and Pictilisib (GDC-0941) PI3K inhibitor reversed these phenotypes. Clinical trials are warranted to examine the efficacy of PI3K inhibition combined with CCRT in cervical cancers.

As mentioned above, *PIK3CA* mutation can be theoretically involved both in radioresistance and cisplatin resistance.^[3,4]^ Our next question was which mechanism is primarily contributing to the observed poor survival in our patients. If radioresistance by mutated *PIK3CA* is the major mechanism, there should have been more recurrences inside the irradiated fields in patients with mutant *PIK3CA* than in those with wild type, but the result was reverse (20% vs 56%; Table 3). Conversely, there were more recurrences outside the irradiated fields in mutant than in wild type (80% vs 52%; Table 3); hence, we presume that cisplatin resistance may be the major mechanism for the survival impact of *PIK3CA* mutation. Patients with cisplatin-resistant tumors will have more recurrences outside the irradiated fields, which should be more critical to prognosis than recurrences inside the irradiated fields, and their recurrence therapies are mostly platinum-based chemotherapies such as paclitaxel and carboplatin. This can explain why wild-type group had better survival even though they included more of persistent diseases with no response to CCRT (10% vs 0%; Table 3) who must have responded more to platinum-based chemotherapy because of platinum-sensitive recurrences. Indeed, by contrast with CSS and OS, when we conducted adjusted multivariate analyses of *PIK3CA* mutation for PFS, only trends without statistical significance were observed (Table 4), most likely reflecting the prognostic impact of *PIK3CA* mutation on sensitivity to recurrence therapies. These findings may suggest that patients would benefit from PI3K inhibitors combined with not only CCRT but also with systemic chemotherapies for recurrence. Further clinical and basic studies are required to clarify this issue.

In conclusion, we have demonstrated here that *PIK3CA* mutation is a significant prognostic factor for poor OS and CSS in cervical cancers treated by CCRT with weekly cisplatin. Together with the previously published findings, the current observations further suggest that molecular inhibitors targeting the PI3K/Akt pathway may improve the outcome by cisplatin-based CCRT in locally advanced cervical cancers harboring *PIK3CA* mutation. We believe that further basic and clinical research will help develop novel treatment strategies and improve the still poor prognosis of patients with locally advanced cervical cancer.

## Author contributions

**Conceptualization:** Hiroyuki Yoshikawa.

**Formal analysis:** Takeo Minaguchi.

**Funding acquisition:** Azusa Akiyama.

**Investigation:** Bouchra Lachkar.

**Methodology:** Takeo Minaguchi, Shuling Liu.

**Project administration:** Takeo Minaguchi.

**Resources:** Azusa Akiyama, Ayumi Shikama, Nobutaka Tasaka, Manabu Sakurai, Sari Nakao, Hiroyuki Ochi.

**Software:** Takeo Minaguchi.

**Supervision:** Toyomi Satoh.

**Validation:** Takeo Minaguchi.

**Visualization:** Bouchra Lachkar.

**Writing – original draft:** Bouchra Lachkar.

**Writing – review & editing:** Takeo Minaguchi, Shuling Liu, Shuang Zhang, Chenyang Xu.

Author name: orcid number
